# Evaluation of the Emergency Care Preparedness of Frontline Junior Doctors: A Training Needs Assessment in Ghana

**DOI:** 10.7759/cureus.107158

**Published:** 2026-04-16

**Authors:** Nkechi O Dike, Jonathan Kajjimu, Nana Serwaa A Quao, Sonia Cobbold, Solomon N Kotey

**Affiliations:** 1 Department of Medical Education and IT, School of Medical Sciences, University of Cape Coast, Cape Coast, GHA; 2 Maersk Clinical Skills Laboratory, School of Medical Sciences, University of Cape Coast, Cape Coast, GHA; 3 Faculty of Medicine, Mbarara University of Science and Technology, Mbarara, UGA; 4 Sparkman Center for Global Health, School of Public Health, The University of Alabama at Birmingham, Birmingham, USA; 5 Department of Emergency Medicine, Korle Bu Teaching Hospital, Accra, GHA; 6 Emergency Medicine Directorate, Komfo Anokye Teaching Hospital, Kumasi, GHA; 7 Department of Epidemiology, School of Public Health and Information Sciences, University of Louisville, Louisville, USA; 8 Division of Environmental Medicine, Department of Medicine, Christina Lee Brown Envirome Institute, University of Louisville, Louisville, USA; 9 Emergency Medicine Unit, The Bank Hospital, Accra, GHA

**Keywords:** clinical competence, emergency medicine, ghana, global health, housemanship, junior doctors, low- and middle-income countries, simulation-based medical education, training needs assessment, undergraduate medical education

## Abstract

Introduction

In low- and middle-income countries (LMICs) like Ghana, junior doctors - house officers and medical officers - serve as the primary frontline providers of emergency care, often in resource-limited settings. Despite their critical role, emergency medicine (EM) training in undergraduate and housemanship curricula remains non-standardized. This study conducted a bottom-up training needs assessment to identify clinical and procedural gaps among junior doctors in Ghana.

Methods

A cross-sectional digital survey was conducted among 75 junior doctors (house officers and medical officers with not more than five years of practice) between October and December 2018. Using 5-point Likert scales, participants self-assessed their comfort with life-saving procedures and their confidence in managing acute medical and trauma presentations. Data were analyzed using descriptive statistics and thematic categorization of qualitative responses.

Results

Although 40% (n = 30) of participants reported managing emergencies “always” in their current roles, only 17.8% (n = 13) felt extremely comfortable as the first-on-call to attend to an emergency or acutely ill patient. While comfort was high for basic tasks like venipuncture (85.9%), it was critically low for advanced procedures; only 8.9% felt comfortable with chest tube insertion, and 87.5% had never performed defibrillation. Confidence was high for managing asthma (90.6%) and hypertensive emergencies (85.0%), but significantly lower for peri-arrest conditions like bradyarrhythmias (70.8% low confidence) and tension pneumothorax. Only 13.5% felt medical school provided excellent preparedness for emergency care, while 100% expressed interest in regular simulation-based training.

Conclusion

A profound mismatch exists between the clinical responsibilities and the formal emergency care training of junior doctors in Ghana. These findings have informed the development of targeted simulation-based training initiatives and have strengthened the case for mandatory EM integration into undergraduate and housemanship curricula across Ghana. To bridge this gap nationally, we recommend that EM be transitioned from an optional to a mandatory component of undergraduate and housemanship training, integrated with decentralized simulation-based medical education.

## Introduction

Emergency care is a fundamental component of effective health systems, delivering time-sensitive interventions for acute illness and injury [1]. Globally, it is recognized as essential to reducing mortality and disability from trauma, cardiovascular emergencies, infections, and other acute conditions [2]. However, many low- and middle-income countries (LMICs) face significant gaps in emergency care capacity, particularly regarding workforce training, infrastructure, and system organization [3,4].

Strengthening emergency care systems is now recognized as a high-priority strategy for achieving universal health coverage and improving population health outcomes, with the World Health Assembly formally identifying workforce development and training as core pillars of this agenda [2,4,5]. Despite this global recognition, structured emergency medicine (EM) training remains limited in many LMICs [6]. In resource-limited settings, frontline providers frequently deliver emergency care without formal specialty training, contributing to significant variability in preparedness and quality of care [7]. In sub-Saharan Africa, while EM has grown as a specialty over the past two decades, workforce shortages and training gaps remain substantial [8-10]. Formalized EM educational infrastructure across the continent continues to evolve unevenly, falling short of the region's high burden of acute disease demands [7-10].

Ghana is among the African countries that have made notable progress in emergency care development. Collaborative initiatives have led to the establishment of EM residency programs, emergency nursing training, and prehospital care, contributing to the emergence of a specialist workforce [1]. However, the number of trained emergency physicians remains insufficient for national coverage and is mainly concentrated in tertiary centers, leaving much of the frontline emergency care across the country delivered by non-specialist providers [11]. This reliance on non-specialized clinicians is further compounded by a critical human resource deficit, evidenced by a very low doctor-to-patient ratio, which places immense strain on the existing healthcare infrastructure [12].

In Ghanaian hospitals, this frontline emergency care is primarily manned and provided by junior doctors, including house officers (HOs) completing their mandatory internship and medical officers (MOs) who have finished their internship but have not yet entered residency [12,13]. During undergraduate medical education, exposure to EM occurs incidentally within broader clinical rotations rather than through a standardized, dedicated EM curriculum [11,14]. While emergency department rotations are available during the two-year housemanship, they are not yet uniformly mandated across all training sites, resulting in wide disparities in clinical exposure and training experiences [15].

In Ghana, medical training primarily follows a direct-entry model, where students enter medical school from secondary education and complete a six-year undergraduate medical program comprising pre-clinical and clinical phases. This is followed by a two-year mandatory internship (housemanship), after which doctors are licensed for independent practice as MOs.

Consequently, these junior doctors are often required to manage critically ill and injured patients, perform life-saving procedures, and make high-stakes decisions in district and peripheral hospitals - typically resource-constrained settings with limited supervision and formal preparation [16]. Similar patterns of training-practice gaps have been documented in other LMIC contexts, where mismatches between clinical responsibilities and structured training raise concerns about provider preparedness and confidence [17,18]. However, despite the central role junior doctors play in emergency care delivery in Ghana, no systematic assessment has documented their perceived preparedness, confidence, or specific emergency care training needs. Such data are essential for developing contextually appropriate curricula, simulation programs, and workforce strengthening interventions.

Therefore, to address this evidence gap, we conducted a training needs assessment using a bottom-up approach. This study allowed junior doctors to identify their own training gaps and self-assess their experience and comfort in managing common emergency presentations and selected procedures.

Study aims

This study aimed to assess experience and self-reported comfort levels in performing selected emergency procedures, and to evaluate confidence in managing common, high-acuity emergency presentations, among junior doctors in Ghana. It further sought to explore their perceptions of educational preparedness derived from undergraduate training, and the potential role of simulation-based medical education (SBME) in bridging identified gaps.

## Materials and methods

Study design and setting

This study was a cross-sectional survey conducted between October and December 2018 to assess junior doctors' perceived training needs in emergency care in Ghana. At the time of the survey, Ghana’s emergency care system was at a critical point, where a formal EM residency program had been established almost a decade earlier, in one of the tertiary centers, Komfo Anokye Teaching Hospital (KATH), and another program was emerging; however, the integration of EM into undergraduate curricula and the mandatory housemanship rotation remained non-standardized. There was limited structured EM exposure during undergraduate medical training and variable learning opportunities in internship emergency rotations. Junior doctors frequently provided frontline emergency care in district, regional, and tertiary hospitals, often in resource-constrained settings and with limited specialist supervision. The study was designed as a “bottom-up” training needs assessment to identify clinical and procedural gaps among frontline junior doctors before developing targeted educational interventions.

Participants

The study population comprised junior doctors practicing in Ghana during the study period. For this study, “junior doctors” were defined as HOs undergoing mandatory supervised internship and MOs who have completed internship and are licensed for independent practice, typically within five years of graduation, and hold a valid Ghana Medical and Dental Council license. We excluded residents, specialists, and MOs with more than five years of post-licensure experience to ensure the data specifically reflected the frontline demographic.

Recruitment and sampling

A convenience sampling approach was employed. The survey link was disseminated via WhatsApp groups (Meta Platforms, Inc., Menlo Park, CA, USA) and professional social media platforms commonly used by HOs and MOs. Peer sharing facilitated further distribution. These platforms provided a direct channel to reach clinicians across various geographical regions, including those in remote district hospitals. Participants represented facilities across different levels of care nationwide. A total of 83 responses were received, of which eight incomplete responses were excluded, resulting in 75 responses included in the final analysis.

Survey instrument

The questionnaire was developed based on competencies and topic areas from the World Health Organization Basic Emergency Care (BEC) course framework, the Ghana HOs’ logbook/manual, and existing emergency care training curricula. Survey items were adapted to reflect emergency presentations and procedures commonly encountered in Ghanaian clinical practice. Content validity was established through expert review by three EM specialists and two experienced MOs (with more than five years of practice) who were not participants in the study.

Variables

The survey collected sociodemographic data and self-reported measures of comfort with emergency procedures using a 5-point Likert scale (1 = very uncomfortable; 5 = very comfortable). Confidence in managing emergency presentations was similarly measured on a 5-point Likert scale (1 = not at all confident; 5 = completely confident) across four domains: emergency procedural skills, common emergency medical presentations, peri-arrest and cardiac arrest situations, and trauma emergencies. Additional variables included interest in simulation-based training and self-identified priority training needs, captured through open-ended responses.

Data collection and analysis

The electronic survey was administered using QualtricsXM (Qualtrics, Provo, UT, USA). Participation was voluntary and anonymous, and completion implied informed consent. An opening information statement outlined the purpose of the study and assured confidentiality. To protect participant privacy, no personally identifiable information was collected.

After excluding eight incomplete entries, 75 responses were included in the final analysis. Quantitative data were initially collected and cleaned in Microsoft Excel (2019; Microsoft Corp., Redmond, WA, USA) following survey administration. For the purposes of this study, the dataset was subsequently re-analyzed in 2025 using Stata version 18.5 SE (StataCorp LLC, College Station, TX, USA), allowing for more robust statistical analysis.

Descriptive statistics (frequencies and percentages) were used to summarize categorical variables, and results were presented in tables and figures. Likert scale responses were treated as ordinal but reported descriptively. Qualitative data from open-ended questions were analyzed using a two-step approach. First, thematic categorization was used to identify common training desires. Second, a word cloud was generated using the Free Word Cloud Generator (freewordcloudgenerator.com) to visually represent the frequency of specific clinical concerns from open-ended responses. Not all survey items were mandatory; therefore, the number of responses (n) varies across variables, and percentages were calculated based on the number of respondents to each specific item.

Ethical considerations

This study was conducted as a training needs assessment for the purpose of curriculum development and quality improvement in medical education under the oversight of the Department of Medical Education and Information Technology at the University of Cape Coast, School of Medical Sciences. Formal institutional review board approval was not required. All participants gave informed consent. All data were handled with strict confidentiality and used solely for the stated educational research purposes.

## Results

Participant characteristics

A total of 75 junior doctors were surveyed, with the majority being under 30 years of age (59, 78.7%), male (39, 52%), and HOs (42, 56%). Most participants worked in urban areas (56, 76.7%) and had graduated from Ghanaian medical schools, with the largest proportion from Kwame Nkrumah University of Science and Technology School of Medical Sciences (29, 38.7%) (Table 1).

**Table 1:** Participants’ demographics UCCSMS: University of Cape Coast School of Medical Sciences; UDS: University of Development Studies; KNUST-SMS: Kwame Nkrumah University of Science and Technology, School of Medical Sciences; UGMS: University of Ghana Medical School *Other category included participants awaiting posting or in transition

Age (years)	Frequency (N = 75)	Percentage (%)
<30	59	78.7
≥30	16	21.3
Sex	Frequency (N = 75)	Percentage (%)
Male	39	52.0
Female	32	42.7
Undisclosed	4	5.3
Cadre	Frequency (N = 75)	Percentage (%)
House officer (HO)	42	56.0
Medical officer (MO)	33	44.0
Medical school attended	Frequency (N = 75)	Percentage (%)
UCCSMS, Ghana	15	20.0
UDS, Ghana	14	18.7
KNUST-SMS, Ghana	29	38.7
UGMS, Ghana	8	10.7
Foreign (China, Cuba, Russia, Ukraine)	9	12.0
Location of practice	Frequency (N = 73)	Percentage (%)
Rural	6	8.2
Peri-urban	11	15.1
Urban	56	76.7
Current place of work	Frequency (N = 74)	Percentage (%)
Teaching hospital	29	39.2
Quasi-government hospital	3	4.1
Ghana health service (district/regional hospital)	20	27.0
Religious-based hospital	7	9.5
Private hospital	9	12.2
Others*	6	8.1

**Figure 1:**
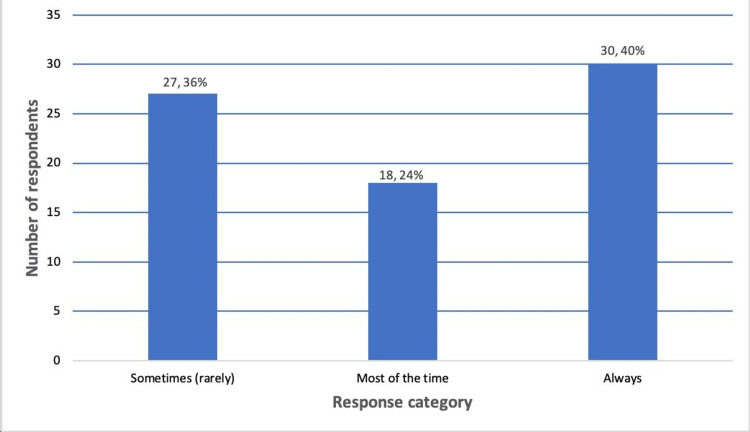
Frequency of managing emergencies or acutely ill patients

**Figure 2:**
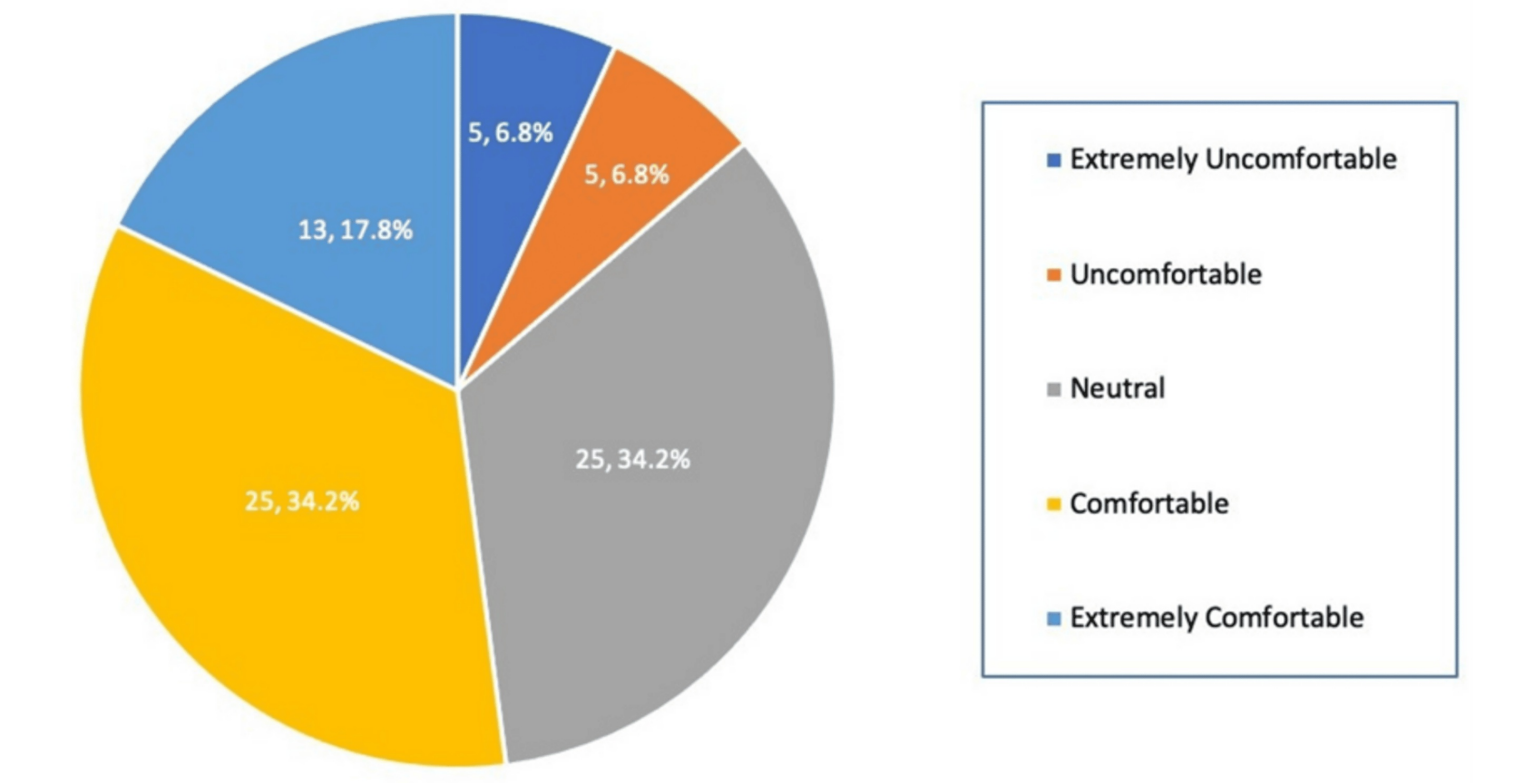
Self-reported comfort level in attending to emergencies or acutely ill patients as the first-on-call clinician

Thirty participants (40%) reported that they always managed emergencies or acutely ill patients (Figure 1), yet only 13 (17.8%) felt extremely comfortable serving as the first-on-call clinician (Figure 2).

Experience and self-reported comfort levels in performing emergency procedures

Participants exhibited wide variation in their experience and comfort levels across procedures. Nearly all respondents (67, 97.1%) had performed venipuncture without supervision, and 85.9% (n = 61) reported being very comfortable performing this procedure. A majority had also performed basic suturing and paracentesis unsupervised; 55 participants (77.5%) had performed basic suturing without supervision, and 40 (65.6%) reported being very comfortable with it. Forty-five participants (64.3%) had also performed paracentesis without supervision, and 41 (65.1%) reported being very comfortable with it.

In contrast, advanced procedures were performed less frequently. Thirty-one respondents (44.3%) had never inserted a chest tube, and only five (8.9%) felt very comfortable with the procedure. Fifty-four participants (77.1%) had no experience of intraosseous cannulation, and only three (6.0%) were very comfortable with it. For defibrillation and synchronized cardioversion, 87.5% (63) and 92.9% (65), respectively, had never performed these procedures. None of the participants had ever performed transcutaneous pacing (Table 2).

**Table 2:** Procedures participants have performed and their comfort level in performing them IV: intravenous; AED: automated external defibrillator

Procedure	Experience				Comfort level					
	N	Never, n (%)	With supervision, n (%)	Without supervision, n (%)	N	Very uncomfortable, n (%)	Uncomfortable, n (%)	Neutral, n (%)	Comfortable, n (%)	Very comfortable, n (%)
Venipuncture/IV cannula insertion	69	0 (0.00)	2 (2.9)	67 (97.1)	71	1 (1.4)	1 (1.4)	3 (4.2)	5 (7.0)	61 (85.9)
Lumbar puncture	70	23 (32.9)	28 (40.0)	19 (27.1)	61	9(14.8)	11 (18.0)	12 (19.7)	15 (24.6)	14 (23.0)
Thoracentesis	70	21 (30.0)	22 (31.4)	27 (38.6)	61	9 (14.8)	10 (16.4)	10 (16.4)	13 (21.3)	19 (31.2)
Paracentesis	70	15 (21.4)	10 (14.3)	45 (64.3)	63	6 (9.5)	2 (3.2)	4 (6.4)	10 (15.9)	41 (65.1)
Chest tube insertion	70	31 (44.3)	30 (42.9)	9 (12.9)	56	15 (26.8)	11 (19.6)	12 (21.4)	13 (23.2)	5 (8.9)
Basic airway techniques/maneuvers	71	15 (21.1)	25 (35.2)	31 (43.7)	59	7 (11.9)	3 (5.1)	13 (18.6)	11 (18.6)	25 (42.4)
Basic suturing skills	71	3 (4.2)	13 (18.3)	55 (77.5)	61	1 (1.6)	2 (3.3)	5 (8.2)	13 (21.3)	40 (65.6)
Basic point-of-care ultrasound	70	25 (35.7)	21 (30.0)	24 (34.3)	50	6 (12.0)	8 (16.0)	12 (24.0)	17 (44.0)	7 (14.0)
Needle compression	69	49 (71.0)	8 (11.6)	12 (17.4)	45	16 (35.6)	7 (15.6)	12 (26.7)	2 (4.4)	8 (17.8)
AED/defibrillation	72	63 (87.5)	7 (9.7)	2 (2.8)	42	24 (57.1)	4 (9.5)	9 (21.4)	3 (7.1)	2 (4.8)
Transcutaneous pacing	72	72 (100.00)	0 (0.00)	0 (0.00)	43	30 (69.8)	3 (7.0)	9 (20.9)	1 (2.3)	0 (0.0)
Synchronized cardioversion	70	65 (92.9)	4 (5.7)	1 (1.4)	45	27 (60.0)	7 (15.6)	9 (20.0)	1 (2.2)	1 (2.2)
Splinting of fractures	71	20 (28.2)	18 (25.4)	33 (46.5)	56	10 (17.9)	2 (3.6)	10 (17.9)	10 (17.9)	24 (42.9)
Intraosseous cannulation	70	54 (77.1)	13 (18.6)	3 (4.3)	50	19 (38.0)	9 (19.0)	13 (26.0)	6 (12.0)	3 (6.0)

Confidence in managing emergency presentations

Most participants generally felt fairly to completely confident managing common emergency presentations, especially hyperglycemic crises (59, 80.9%), hypertensive emergencies (62, 85.0%), and acute asthma exacerbations (63, 90.6%). In contrast, confidence was notably lower for peri-arrest and cardiac arrest conditions; 51 (70.8%) reported not at all or only slight confidence in managing bradyarrhythmias, and 49 (68.1%) reported the same for tachyarrhythmias.

Among trauma-related emergencies, confidence was lowest for hemothorax and tension pneumothorax, with 31 (43.1%) and 26 (35.6%) participants, respectively, reporting not at all or only slightly confident in managing these conditions. Confidence was comparatively higher for fractures and joint dislocations, though variable across other trauma presentations (Table 3).

**Table 3:** Self-reported confidence in managing emergency medical and trauma presentations MI: myocardial infarction; DKA: diabetic ketoacidosis; HHS: hyperosmolar hyperglycemic state; PEA: pulseless electrical activity; V.Fib: ventricular fibrillation; pVTach: pulseless ventricular tachycardia

Conditions	Frequency (N)	Not at all confident, n (%)	Slightly confident, n (%)	Somewhat confident, n (%)	Fairly confident, n (%)	Completely confident, n (%)
Common emergency presentations						
Acute chest pain (excluding MI)	72	2 (2.8)	8 (11.1)	13 (18.1)	31 (43.1)	18 (25.0)
Acute MI	72	4 (5.6)	6 (8.3)	13 (18.1)	33 (45.8)	16 (22.2)
Acute shortness of breath	73	1 (1.4)	4 (5.5)	9 (12.3)	40 (54.8)	19 (26.0)
Altered mental status	71	6 (8.5)	7 (9.9)	17 (23.9)	32 (45.1)	9 (12.7)
Seizures	73	3 (4.1)	4 (5.5)	10 (13.7)	29 (39.7)	27 (37.0)
Anaphylaxis	73	3 (4.1)	9 (12.3)	11 (15.1)	35 (48.0)	15 (20.6)
Hyperglycemic crisis (DKA/HHS)	73	2 (2.74)	6 (8.2)	6 (8.2)	18 (24.7)	41(56.2)
Hypertensive emergencies	73	4 (5.5)	3 (4.1)	4 (5.5)	15 (20.6)	47 (64.4)
Acute asthmatic attack	74	1 (1.4)	4 (5.4)	2 (2.7)	23 (31.1)	44 (59.5)
Peri-arrest and cardiac arrest presentations						
Bradyarrhythmia	72	24 (33.3)	27 (37.5)	9 (12.5)	12 (16.7)	0 (0.0)
Tachyarrhythmia	72	21 (29.2)	28 (38.9)	9 (12.5)	14 (19.4)	0 (0.0)
Shock (hypoperfusion)	72	0 (0.0)	2 (2.8)	13 (18. 1)	23 (31.9)	34 (47.2)
Opioid overdose	73	13 (17.8)	16 (21.9)	18 (24.7)	19 (26.0)	7 (9.6)
Hypoglycemia	73	1 (1.4)	1 (1.4)	3 (4.1)	12 (16.4)	56 (76.7)
Cardiac arrest (PEA/asystole)	73	22 (30.1)	21 (28.8)	8 (11.0)	14 (19.2)	8 (11.0)
Cardiac arrest (V.Fib/pVTach)	71	32 (45.1)	16 (22.5)	13 (18.3)	7 (9.9)	3 (4.2)
Trauma emergencies						
Tension pneumothorax	73	14 (19.2)	12 (16.4)	23 (31.5)	15 (20.6)	9 (12.3)
Hemothorax	72	12 (16.7)	19 (26.4)	17 (23.6)	18 (25.0)	6 (8.3)
Uncontrolled bleeding	72	5 (6.9)	15 (20.8)	20 (27.8)	24 (33.3)	8 (11.1)
Fractures	74	8 (10.8)	7 (9.5)	12 (32.4)	24 (32.4)	23 (31.1)
Joint dislocations	71	10 (14.1)	11 (15.5)	16 (22.5)	26 (36.6)	8 (11.3)
Traumatic head injuries	71	11 (15.5)	10 (14.1)	18 (25.4)	24 (33.8)	8 (11.3)

Perceptions of educational preparedness and simulation-based emergency care training

Only 10 (13.5%) rated their medical school preparation for emergency management as excellent. Four participants (5.4%) rated their preparedness from medical school to perform life-saving emergency procedures as “excellent.” Almost all participants (73, 98.7%) considered the incorporation of simulations for emergency cases and procedures into the medical school curriculum to be “very useful.” All participants expressed interest in participating in regular simulation-based training (Table 4).

**Table 4:** Perceptions of educational preparedness and simulation training in the management of emergencies

Perception	n (%)
Preparedness level from medical school to manage emergencies	
Poor	5 (6.8)
Somewhat good	18 (24.3)
Good	24 (32.4)
Very good	17 (23.0)
Excellent	10 (13.5)
Preparedness level from medical school to perform life-saving emergency procedures	
Poor	14 (18.9)
Somewhat good	22 (29.7)
Good	19 (25.7)
Very good	15 (20.3)
Excellent	4 (5.4)
Perception about incorporating simulations of emergencies and procedural skills into medical school curriculum	
Very useful	73 (98.7)
Slightly useful	1 (1.4)
Not useful	0 (0.0)
Can clinical practice alone adequately improve the management of emergencies over time?	
Strongly disagree	5 (6.8)
Somewhat disagree	19 (25.7)
Neutral (neither agree nor disagree)	12 (16.2)
Somewhat agree	24 (32.4)
Strongly agree	14 (18.9)
Can clinical practice alone adequately improve competency in correctly performing emergency procedures over time?	
Strongly disagree	5 (6.8)
Somewhat disagree	15 (20.3)
Neutral (neither agree nor disagree)	9 (12.2)
Somewhat agree	26 (35.1)
Strongly agree	19 (25.7)
Are you interested in regular simulations of clinical scenarios to improve your skills in the management of emergencies and procedures?	
Yes	74 (100.0)
No	0 (0.0)

**Figure 3:**
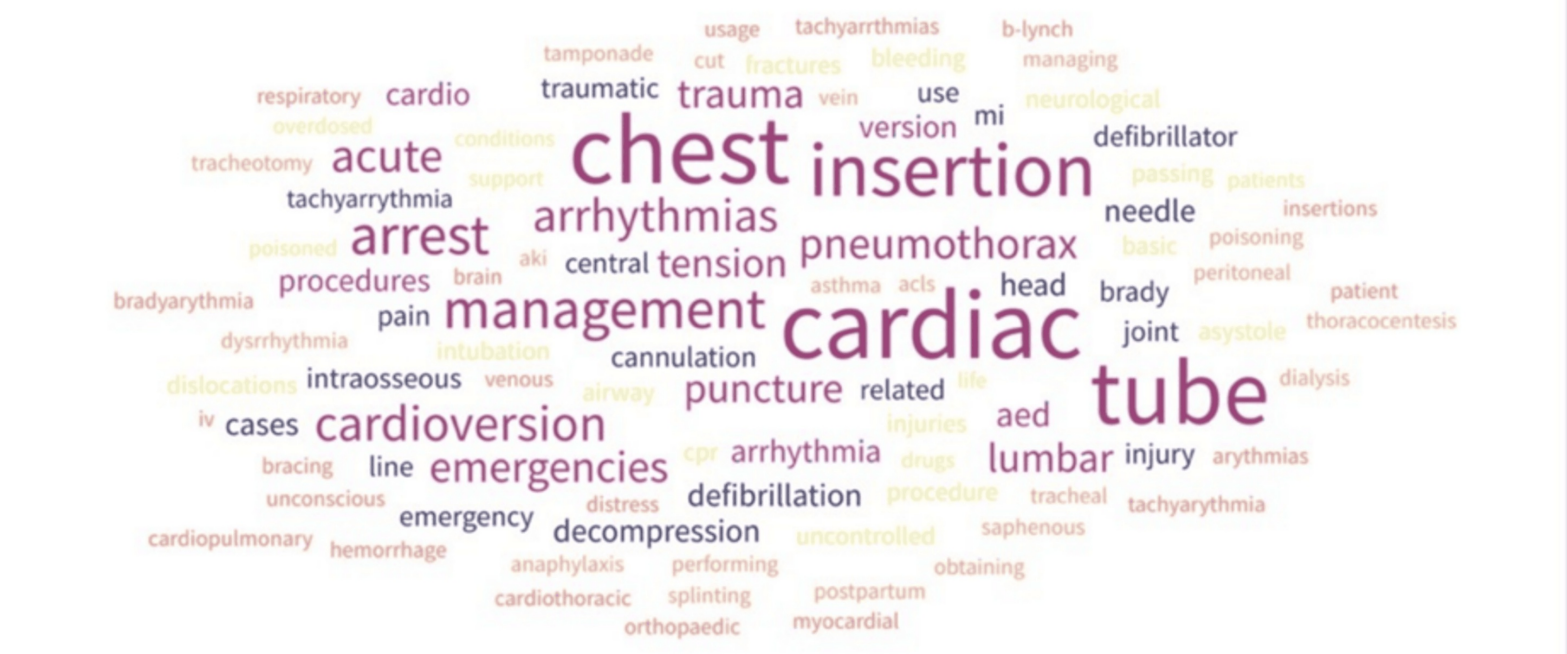
Emergency management training needs (cases and procedures) identified by participants Word cloud generated by the authors using Free Word Cloud Generator (freewordcloudgenerator.com)

When asked about preferred training areas, suggested topics included management of cardiac arrest and life support, airway and ventilation management, trauma emergencies, neurological emergencies, poisoning, vascular access, and obstetric and gynecological emergencies, as shown in Figure 3. Overall, many responses combined a clinical presentation (e.g., cardiac arrest) with their corresponding procedural interventions (e.g., defibrillation and chest tube), perhaps reflecting a need for simulation to integrate procedural training within clinical scenarios.

## Discussion

This training needs assessment among junior doctors in Ghana reveals a consistent and important pattern; comfort and confidence were relatively higher for basic, routinely performed clinical tasks, but fell sharply for advanced procedures and high-acuity emergency presentations. These findings point to a clear mismatch between the clinical responsibilities junior doctors are expected to carry and the emergency care training they actually receive during undergraduate medical education and housemanship [16-18].

For the purpose of this study, basic procedures refer to commonly performed, low-risk clinical tasks such as venipuncture, suturing, and paracentesis, while advanced or critical procedures include less frequently performed, high-acuity interventions such as chest tube insertion, intraosseous access, and defibrillation, which are typically required in life-threatening emergencies.

The observed gradient in procedural comfort is not unique to Ghana. Similar gaps have been reported in other settings, where junior clinicians demonstrate limited preparedness for high-acuity emergencies and variable exposure to advanced procedures such as resuscitation and critical care interventions [19-22]. Junior doctors in Malaysia, Ethiopia, and Canada have similarly reported stronger familiarity with basic procedures and notable gaps in advanced procedural skills [19-21]. By contrast, EM trainees in Saudi Arabia reported higher confidence across advanced procedures [22]. This difference likely reflects that they had more structured, supervised procedural exposure within formalized training programs. The key takeaway for Ghana is straightforward: without deliberate exposure to advanced procedures within the curriculum, routine clinical work alone will not prepare junior doctors for high-stakes interventions. The finding that 87.5% of participants had never performed defibrillation is perhaps the clearest illustration of this gap, given that many of these same doctors were already serving as first-on-call for emergency presentations.

Confidence in managing emergency presentations followed a similar pattern. Participants reported relatively high confidence for common non-traumatic conditions such as asthma exacerbations and hypertensive emergencies, which likely reflects more frequent exposure during routine clinical duties. Confidence was notably lower, however, for peri-arrest conditions, arrhythmias, and trauma emergencies. These are precisely the scenarios where delayed or incorrect management carries the highest risk of death or disability [2]. This is broadly consistent with findings from other developing-country settings, where low baseline performance in critical emergency scenarios has been documented before targeted educational interventions [23]. One important caveat worth noting is that self-reported confidence does not always reflect actual clinical competence. Future studies in this context should incorporate objective measures, such as simulation-based performance assessments or structured clinical examinations, to provide a more reliable picture of preparedness.

The near-universal endorsement of SBME by participants is a significant finding in itself. The fact that 100% of respondents expressed interest in regular simulation training, and that 98.7% rated its incorporation into the medical curriculum as very useful, suggests more than a preference. It suggests that junior doctors are genuinely aware of the gaps in their preparation and are asking for a safer environment in which to develop these skills. This aligns well with the broader evidence base, which consistently shows that simulation improves procedural competence, clinical reasoning, and skill retention, particularly for high-risk, low-frequency events where traditional bedside learning is limited or ethically difficult to justify [24-26]. The qualitative responses further reinforced this, with participants identifying integrated training needs by pairing emergency presentations with their corresponding procedural interventions, therefore indicating a desire not just for isolated procedural practice but for integrated, context-driven simulation training.

These findings align with and have contributed to emerging educational responses, even if incrementally. The specific procedural and clinical gaps identified, combined with the near-unanimous call for simulation, informed the development of targeted training initiatives at both undergraduate and early postgraduate levels. At the University of Cape Coast School of Medical Sciences, high-fidelity simulation was integrated into the undergraduate curriculum in a structured, progressive manner, introducing resuscitation skills early and building toward advanced life support by the final clinical year, with procedural competencies assessed within the Objective Structured Clinical Examinations (OSCEs). However, this needs to be scaled up formally across curricula in all medical schools in Ghana. Additionally, as awareness of the need for experiential emergency training grows, simulation-based continuing professional development (CPD) initiatives are now being piloted across different regions [27].

In direct response to the training needs identified in this study, an online training platform was piloted for general practicing doctors, particularly those in remote and underserved areas. Selected emergency topics are delivered in a bite-sized, longitudinal format using case-based teaching, grand rounds, gamification, and virtual simulation, allowing doctors in peripheral settings to engage with clinical decision-making in a flexible and accessible way.

At the policy level, these findings support ongoing discussions around the growing evidence that EM must be made a mandatory component of both undergraduate and housemanship training in Ghana. The Medical and Dental Council of Ghana's recent introduction of an optional EM rotation during housemanship is a step in the right direction. However, its reach remains limited, with the rotation currently available in just over 20 hospitals, each with dedicated EM specialists [15]. Its zero-sum structure also creates a difficult trade-off, requiring HOs to forfeit a core rotation in internal medicine or surgery to pursue EM. This means it cannot yet serve as an equitable or consistent solution nationwide. Since the majority of emergency care in Ghana is delivered by non-specialist junior doctors, often in peripheral and district hospitals with limited supervision, EM training needs to be treated as a foundational clinical competency for every graduating doctor, not as a specialty elective.

These developments demonstrate that, when bottom-up needs assessment data are collected systematically and honestly, they can serve as a credible basis for curriculum reform [17]. While these efforts are still in their early stages, they reflect a broader recognition that sustainable emergency care training in Ghana must account for geography, resource constraints, and the realities of the frontline workforce.

Limitations

This study has several limitations worth acknowledging. The relatively small sample size and use of convenience sampling via digital platforms may limit generalizability, particularly to junior doctors with limited digital access. This approach may also introduce digital sampling bias, as a higher proportion of respondents were based in urban settings. In addition, the use of self-reported measures of comfort and confidence, while valuable indicators of perceived preparedness, does not directly equate to objectively measured clinical competence.

The data were collected in 2018 and therefore reflect a pre-intervention baseline of emergency care preparedness prior to subsequent educational reforms. While this provides an important reference point for evaluating system-level changes over time, the findings may not fully represent current conditions. Future comparative and longitudinal studies are needed to assess evolving trends in workforce capability following these interventions. Finally, as this was an exploratory study, no formal sample size or power calculation was conducted; however, the findings provide important preliminary insights to inform targeted educational strategies and further research.

## Conclusions

This study confirms a meaningful mismatch between the clinical responsibilities of junior doctors in Ghana and the emergency care training they receive during undergraduate medical education and housemanship. Comfort and confidence were consistently lower for advanced procedures and high-acuity presentations, while perceptions of preparedness from medical school were poor across the board. The near-universal interest in simulation-based training signals that junior doctors are both aware of these gaps and are motivated to address them.

To close this gap, EM should be formally introduced into medical school curricula across Ghana. We also recommend that EM be transitioned from an optional rotation to a mandatory component of both undergraduate and housemanship curricula, accompanied by the decentralized integration of SBME that can realistically reach doctors in peripheral and resource-limited settings, as continuous professional development programs and in-service training. These recommendations are grounded in direct evidence from the frontline workforce they are meant to serve, and this study makes the case for routine bottom-up needs assessment as a core part of curriculum development in Ghana and similar settings.

## Appendices

Training Needs Assessment Questionnaire (Complete Instrument)

Section A: Demographics

**Table 5:** Demographics UCCSMS: University of Cape Coast School of Medical Sciences (UCCSMS), Ghana; UDS: University of Development Studies, Ghana; KNUST: Kwame Nkrumah University of Science and Technology, School of Medical Sciences, Ghana; UGMS: University of Ghana Medical School, Ghana

Question No.	Question	Response options
Q1	Age (years)	___________
Q2	Gender	Male/Female/Prefer not to say
Q3	Medical school	UCCSMS/UDS/KNUST/UGMS/Other (specify country): ___________
Q3	Year of graduation	___________
Q4	Current position (house officers)	House officer (1st year)/Senior house officer (2nd year)
Q4	Departments rotated	Internal medicine/Pediatrics/Surgery/Ob-Gyn/Psychiatry/Anesthesia
Q5	Current position (medical officers)	1st year medical officer/2nd year medical officer
Q5	Department	___________
Q6	Current workplace	Teaching hospital/Quasi-government hospital/Ghana health service (district/regional)/CHAG (religious-based)/Private hospital/Other: ___________
Q7	Location of practice	Rural/Peri-urban/Urban

Section B: Frequency of Managing Emergencies and Comfort Level

**Table 6:** Frequency of managing emergencies and comfort level as first-on-call Scale for Q9: 1: Extremely uncomfortable; 5: Extremely comfortable

Question No.	Question	Response options
Q8	How often do you manage emergencies or acutely ill patients?	Never/Sometimes/Most of the time/Always
Q9	How comfortable are you as first-on-call for emergencies? (1–5)	1/2/3/4/5

Section C: Procedural Experience

Q10. Please answer these questions concerning the listed procedures

Q10.1: Have you performed this procedure before? (1: Never; 2: With supervision; 3: Without supervision)

Q10.2: Rate your comfort level performing each procedure (1: Very uncomfortable; 2: Slightly uncomfortable; 3: Neutral; 4: Slightly comfortable; 5: Very comfortable)

**Table 7:** Procedural experience and comfort level in performing them IV: intravenous; AED: automated external defibrillator

Procedure	Experience (1–3)	Comfort (1–5)
Venipuncture/IV cannula insertion	1/2/3	1/2/3/4/5
Lumbar puncture	1/2/3	1/2/3/4/5
Thoracentesis	1/2/3	1/2/3/4/5
Paracentesis	1/2/3	1/2/3/4/5
Chest tube insertion	1/2/3	1/2/3/4/5
Basic airway technique/maneuver	1/2/3	1/2/3/4/5
Basic suturing skills	1/2/3	1/2/3/4/5
Basic point-of-care ultrasound	1/2/3	1/2/3/4/5
Needle decompression	1/2/3	1/2/3/4/5
AED/defibrillation	1/2/3	1/2/3/4/5
Transcutaneous pacing	1/2/3	1/2/3/4/5
Synchronized cardioversion	1/2/3	1/2/3/4/5
Splinting of fractures	1/2/3	1/2/3/4/5
Intraosseous cannulation	1/2/3	1/2/3/4/5

Section D: Confidence in Managing Common Presentations

Q11. Please rate your confidence in managing the listed common presentations (Scale: 1: Not confident at all; 2: Slightly confident; 3: Somewhat confident; 4: Fairly confident; 5: Completely confident)

**Table 8:** Confidence in managing common presentations MI: myocardial infarction; DKA: diabetic ketoacidosis; HHS: hyperosmolar hyperglycemic state

Clinical presentation	1	2	3	4	5
Acute chest pain (excluding MI)					
Acute myocardial infarction					
Acute shortness of breath					
Altered mental status					
Seizures					
Anaphylaxis					
Hyperglycemic crisis (DKA/HHS)					
Hypertensive emergencies					
Acute asthmatic attack					

Section E: Confidence in Peri-Arrest and Arrest Situations

Q12. Please rate your confidence in managing the listed peri-arrest and cardiac arrest presentations (Scale: 1: Not confident at all; 2: Slightly confident; 3: Somewhat confident; 4: Fairly confident; 5: Completely confident)

**Table 9:** Confidence in peri-arrest and cardiac arrest situations PEA: pulseless electrical activity; V.Fib: ventricular fibrillation; pVTach: pulseless ventricular tachycardia

Clinical scenario	1	2	3	4	5
Bradyarrhythmia					
Tachyarrhythmia					
Shock (hypoperfusion/hypotension)					
Opioid overdose					
Hypoglycemia					
Cardiac arrest (PEA/asystole)					
Cardiac arrest (V.Fib/pVTach)					

Section F: Confidence in Trauma Emergencies

Q13. Please rate your confidence in managing the listed trauma emergencies (Scale: 1: Not confident at all; 2: Slightly confident; 3: Somewhat confident; 4: Fairly confident; 5: Completely confident)

**Table 10:** Confidence in trauma emergencies

Trauma presentation	1	2	3	4	5
Tension pneumothorax					
Hemothorax					
Uncontrolled bleeding					
Fractures					
Joint dislocations					
Traumatic head injuries					

Section F: Preparedness and Perceptions

Q14. On a scale of 1 to 5 (1: Poor; 5: Excellent), how will you rate your preparedness level from medical school for

Q14.1: Medical emergencies

Q14.2: Performing life-saving emergency procedures

Q15. What is your perception about incorporating simulations of emergencies and procedural skills into the medical schools' curriculum? (Scale: 1: Very useful; 2: Slightly useful; 3: Not useful)

Q16. Do you think that clinical practice alone, done over a period of time, can adequately improve (Scale: 1: Strongly disagree; 2: Somewhat disagree; 3: Neutral; 4: Somewhat agree; 5: Strongly agree)

Q16.1: Your management of emergencies

Q16.2: Your competency in correctly performing emergency procedures

Q17. Will you be interested in regular simulation sessions to improve your skills in the management of emergencies and procedures? (Scale: 1: No; 2: Yes)

Q18. Please indicate any two (2) areas each in which you would like to be trained in the management of emergencies (2 cases and 2 procedures)

**Table 11:** Preparedness and perceptions

Question No.	Question	Response options
Q14.1	Preparedness to manage emergencies (1–5)	1/2/3/4/5
Q14.2	Preparedness to perform life-saving procedures (1–5)	1/2/3/4/5
Q15	Perception of simulation in curriculum	1 (Very useful)/2 (Slightly useful)/3 (Not useful)
Q16.1	Clinical practice alone improves emergency management	1/2/3/4/5
Q16.2	Clinical practice alone improves procedural competency	1/2/3/4/5
Q17	Interest in simulation training	No/Yes
Q18	Areas of interest for training (2 cases, 2 procedures)	__________________________________________
